# Acceptability of a parent-focused program for physical literacy development among parents and children during the COVID-19 pandemic: A qualitative study

**DOI:** 10.3389/fpubh.2022.924434

**Published:** 2022-09-15

**Authors:** Amy S. Ha, Taoran Zeng, Qing He, Cecilia H. S. Chan, Ying Fung, Johan Y. Y. Ng

**Affiliations:** Department of Sports Science and Physical Education, The Chinese University of Hong Kong, Shatin, Hong Kong SAR, China

**Keywords:** online-learning, family, implementation outcomes, social cognitive theory, qualitative research

## Abstract

**Background:**

A 3-month parent education program was designed and implemented in Hong Kong to improve physical literacy (PL) among primary school children and their parents during the COVID-19 pandemic. This study aims to probe into the acceptability of the intervention from parental perspectives, providing more insights for future implementation.

**Methods:**

Following the 3-month parent-focused PL program, 16 parents (mean age = 43.73 years, 15 mothers) were recruited to participate in semi-structured individual interviews in June 2021. Interviews were analyzed using thematic analysis in NVivo 12. Two coders analyzed interview transcripts deductively based on the interview guide and social cognitive theory (SCT).

**Results:**

Three themes were identified that captured the acceptability of the program: (1) Addressing needs through appropriate delivery enhances acceptability of intervention; (2) Positive behavioral and psychological changes to families; (3) Appropriate support of environmental factors can enhance the sustainability of program effects. The high levels of acceptability were attributable to the fact that parents were able to learn and be a gatekeeper to impact their children positively. Moreover, the design and contents of the program were appropriate for the participating parents because the program provided adequate learning resources and interactive learning support without being overly rigid, and the online learning mode was feasible and convenient. Consequent to their participation in the program, parents expressed that they became more aware of the importance of PL, established physical activity routines with their children, and modified their parenting styles which resulted in improved parent-child relationships.

**Conclusions:**

The current study provided evidence that engaging parents in the intervention was feasible and acceptable approach in supporting both parents and children to establish physically active routines in the family setting. The insights gained regarding the appropriateness and acceptability of the program in this specific context may be used to inform the design, implementation and sustainability of other parent-focused PL programs.

## Introduction

Physical literacy (PL) is defined as “the motivation, confidence, physical competence, knowledge and understanding to the value and take responsibility for engagement in physical activity (PA) for life” (1). Physical literacy is an antecedent to PA participation (2, 3). Researchers suggested that regular participation in physical activities (PA) is beneficial to both physical and mental health, such as reducing the risk of chronic diseases and the incidence of mental health disorders (4, 5). Improvements in PL *via* intervention should fundamentally help sustain the positive effects of PA interventions.

While physical education is essential in helping children gain knowledge of the importance of PA, and learn fundamental movement skills and other sports skills (to build competence) from teachers or sports coaches, parents' roles in the development of PL for young kids and school-aged children are equally important (3). Evidence indicates that support in the home environment and parenting behaviors are crucial in shaping and promoting children's PA (6). Parents typically play an essential role in children's growth development and in shaping their PA behaviors (7) and hence the development of PL. The improvement of parents' PL could help sustain the effects of PA interventions targeting children or families. Hence parent-focused interventions hold promise for achieving health impact on both parents and children (8). However, in Hong Kong, parents put a large emphasis on children's academic development (9) and the rate of inactivity in children and parents is high (10). Moreover, since the outbreak of the COVID-19 pandemic in 2019, the everyday lives of people around the globe had been greatly affected. Schools and PA interest classes were suspended for a prolonged period. Parents started to work from home while children were having online classes at home. In turn, children's PA levels dropped significantly (11). There was an urgent need and a good opportunity to improve parents' PL. Therefore, we developed and implemented a 3-month parent-focused PL program during the COVID-19 pandemic.

Past studies on PA interventions in the family context (8, 12, 13) were mainly quantitative and were unable to capture the underlying processes or mechanisms in terms of why they might be effective (14). Thus, we adopted a qualitative approach in this study, aiming to explore the acceptability of the parent-focused program and related factors of sustainability of the intervention. We applied social cognitive theory (SCT) (15), which posits that individuals behaviors and environments are interrelated, to explore participants' perceptions of the program, facilitators and barriers to PA in the family context before and during the COVID-19 pandemic. Through individual interviews with participants of the program, we aimed to solicitate their views and feedback to specific components of the intervention, and in turn to explain these processes in light of SCT. This study contributes to the understandings of the acceptability and the importance of a parent-focused PL program, which would benefit the nurturance of familial healthy lifestyles and the sustainability of PA interventions in the family context.

## Methods

### Study design

A qualitative study design was employed in this study to explore parents' acceptability toward the designed intervention contents through verbal interviews.

### Participants and procedures

The parent-focused program described in this study is an initiative under the broader Fun to Move@JC project (10, 16). Fun to Move@JC is a school-based multi-component project launched in 2017 designed to enhance PA of primary school students and their parents. Only families who have taken part in the broader project was invited to take part in the PL intervention program. All parents who completed the PL program were invited to take part in a qualitative research study. Parents were told that they and their children would be interviewed by project staff if they agreed to take part. A total of 16 parents of 16 schoolchildren from 12 Hong Kong primary schools responded to our invitation and took part in this study. Respondents from all four major geographical areas in Hong Kong were included to ensure sample representativeness. Parent participants included mothers (*n* = 15) and fathers (*n* = 1), with a mean age of 43.73 years. Most parents (69%, *n* = 11) were housewives, and 75% of them had a family monthly income of HK$20,000-$60,000 (the median household income of Hong Kong is around HK$28,000). The majority spoke Cantonese at home (87.5%, *n* = 14; Table 1).

**Table 1:** Demographic information of participants.

**Characteristics**	**Mean (SD) or *N* (%)**
**Gender**	
Male	1 (6)
Female	15 (94)
Age (years)	43.73 (4.62)
**Educational attainment**	
Up to lower secondary school	1 (6)
Upper secondary school	8 (50)
Associate degree or higher diploma	4 (25)
Undergraduate degree	3 (19)
**Employment status**	
Housewife	11 (69)
Employed, part-time	1 (6)
Employed, full-time	4 (25)
**Language spoken at home**	
Cantonese	14 (87.5)
Mandarin	2 (12.5)
**Number of children**	
1	8 (50)
2	8 (50)
**Family monthly income (HK$)**	
<20,000	2 (12.5)
20,000–60,000	12 (75)
>60,000	2 (12.5)

Individual interviews were arranged with each participating family. Two members of the research team conducted the interviews separately in participants' first language (i.e., Cantonese). Due to the pandemic-related social distancing measures enforced, interviews were conducted *via* Zoom or phone based on participants' preferences and convenience. Each interview lasted ~30 min. At the beginning of each interview, participants consented to the session being audio-recorded. Each parent completed a questionnaire after the interview to collect demographic information, including age, marital status, educational background, monthly family income, and the number of children in their household. After the completion of interviews with this group of participants, there was evidence of data saturation (17). As such, we did not extend our efforts to recruit additional participants to the study.

### Intervention contents

The contents of the parent-focused program were designed to improve all facets (i.e., knowledge and understanding, motivation and self-confidence, motor competence, and activity behaviors) of PL in parents. Details of the intervention contents are presented elsewhere (16). In brief, the main intervention was delivered through six 60-min interactive workshops, with each being 2 weeks apart. Hence the total duration of the intervention was approximately three calendar months. Workshops were conducted online amid pandemic-related social distancing measures that were in force. Nonetheless, this delivery approach also allowed parents from all geographical regions of Hong Kong to attend simultaneously. Various topics (e.g., cognitive development, mental health and parent-child relations) related to PA were presented by the lead author during the first halves of workshop sessions. The objectives of these sessions were to enhance parents' awareness, knowledge, and understanding of PA, and to motivate and instill confidence in them to remain physically active with their children.

The final 30 min of each workshop was an activity lesson led by coaches trained by the authors, where parents were invited to do various activities under the guidance of coaches trained by the authors. Coaches demonstrated and led participants to exercises related to physical fitness and fundamental movement skills, and provided recommendations in terms of how parents could lead their children to do similar activities at home. Through these instructions, we aimed to improve parents' competence in fitness and motor skills, and provided more co-activity ideas for parents and children.

### Data analysis

All interviews were audio-recorded and transcribed verbatim. Interview transcripts went through member checking to ensure trustworthiness (18) before being analyzed using the inductive-deductive hybrid thematic analysis (19) *via* NVivo Version 12. First, a preliminary codebook was developed based on the interview guide (20). Two authors worked as coders in the data analysis phase. First, they independently analyzed all the transcripts with the preliminary codebook, adding codes inductively to capture phenomena not included in the preliminary codebook. Third, they compared coding and came to a consensus on all discrepant codes to be added to the codebook. Fourth, codes were connected to identify themes, they made a consensus on the themes and sub-themes, and the final codebook was sent to all the authors for review. The final set of codes and corresponding descriptions for the themes and sub-themes are presented in Table 2.

**Table 2:** Outline of theme, sub-theme, and codes used in qualitative analysis.

**Theme**		**Sub-theme**	**Description of codes**
**1. Addressing needs through appropriate delivery enhances the acceptability of the intervention**		Motivation and expectation toward the program	1. School recommendation 2. Do more PAs during the pandemic
		Acceptability of the program	1. Absorb knowledge from the workshop 2. Scope and depth of content of workshops 3. Arrangement and format 4. Accessibility of learning recourses
		Online delivery mode	Positive attitudes: Avoid infection Flexible Convenient Time-saving
		Suggestions and improvements	1. Extend the duration of the workshop 2. Generalize the program to broader families 3. Hybrid mode to deliver the workshop 4. Involvement of children
**2. Positive behavioral and psychological changes to families**		Perceived behavioral and psychological change	1. More PA/Co-PA time 2. Better quality of sleep 3. Reduce screen time 4. Embedded the rationale of the PA knowledge to daily activities
		Long-term effect	1. Establishing PA habits and family routine 2. Be more aware of the importance of exercise
		Parenting behaviors	1. Parenthood 2. Reflection on parenting style
**3. Appropriate support of environmental factors can enhance the sustainability of program effects**	**Social cognitive factors**	Attitudes and values toward PA	1. Perceived PA benefits (physical & mental) 2. Perceived co-activity benefits
		Parent self-efficacy to PA	1. PA/exercise habits 2. Perceived PA barriers
		Parent self-regulation to PA	1. PA goal setting 2. Monitor PA
	**Physical environment factors**	Hong Kong built environment	1. Sports facilities/venues accessibility 2. Transportation 3. Population density
		Home environment	1. Limited space 2. Unfavorable home setting

## Results

Elaborations and illustrative quotations to support our analyses are presented below. All transcriptions presented were translated from Cantonese, which was the language in which the interviews were conducted and transcribed.

### Addressing needs through appropriate delivery enhances the acceptability of the intervention

The program delivered PL knowledge to parents, which largely addressed their needs during the pandemic. Parents mentioned that they were initially motivated to participate because they were affected by the epidemic and realized they needed to do more exercise.

“*Lectures on PA have been scarce [in HK]. As this parent program was launched, although the time did not allow me to fully participate [in all sessions], I thought it was good to attend at least the first two workshops.”* (Interviewee 2, Mother)

Parents also expressed satisfaction with the program's content and organization in various ways, including the acquisition of theoretical knowledge, the interactive experience in the sessions, the utility of the learning resources, and the online learning mode. The theoretical knowledge set the fundament of their PL. For example, one mother noted:

“*I believe it is important for us to understand why PL is beneficial to children and what problems can arise if we do not engage in PA. We probably would not know as much as we do without the theoretical courses, and the sharing sessions also include educational knowledge, such as to teach children to be persistent in PA and how to manage time to implement the plan effectively*.” (Interviewee 1, Mother)

To address their needs precisely, we adopted parents' suggestions and recommendations from the first two workshops in subsequent workshops that contributed to parents' satisfaction. One parent stated that:

“*I suggested in the third or fourth class that there could be more interactive sessions, and [the organizers] really added more interaction in the next workshop*.” (Interviewee 3, Mother)

Parents praised the combination of educational content with the activity sessions. They learned some feasible ways to do PA at home at the workshops and then applied them in daily life.

“*I learned (from the workshop) that exercising is not limited to outdoor types. Many exercises can be conducted within limited space at home.”* (Interviewee 12, Mother)

“*You used simple materials, e.g., plastic bottles and towels, to do PA (at the workshop). After the workshop, we engaged in these activities, e.g., using towels to stretch, which we would not do before attending the workshop.”* (Interviewee 11, Mother)

The learning materials we designed, such as the worksheets, also helped parents improve memory and review workshop knowledge, which facilitated the sustainability of the program.

“*Some of the questions were about time management, and the graphs allowed us to self-regulate how much time we spent each day doing exercise in our daily lives*.” (Interviewee 1, Mother)

The online delivery mode of the program was convenient for parents. They could arrange their time flexibly and keep social distance from others during the pandemic, though it also has some disadvantages compared to face-to-face modes.

“*The online mode saved transportation time….The face-to-face mode allows people to interact closely with each other, especially for children. The atmosphere would be happier. But it would be the same for adults.”* (Interviewee 4, Mother)

The online delivery mode ensured their participation during the specific time and is an option to improve the sustainability of the program in the future. One father noted:

“*If there are future opportunities to conduct related activities, I believe the learning format can be alternated between face-to-face and online classes*.” (Interviewee 13, Father)

### Positive behavioral and psychological changes to families

This program applied a more holistic approach in the intervention, i.e., focusing on PL and not just PA, so participants could be benefited on physical, cognitive and affective levels. Specifically, parents were not just taught about how to do specific PA with children, but also about the importance of PA on other aspects, such as children cognitive development, parent-child relations. Intervention contents were also specifically designed to encompass each area of PL. Parents elaborated on three main aspects of changes after engaging in the program, namely perceived behavioral changes, psychological changes, and reflections on parenting behaviors. Health and wellbeing, psychological symptoms (e.g., depression, anxiety), changes in sleep pattern and weight, more PA time, and less screen time were mentioned in terms of behavioral and psychological change. The PL knowledge was considered interesting and down to earth so could be applied in daily life and changes be seen, in both parents and children.

“*After participating in the workshops, I was particularly impressed by the workshop on family routines, which is the starting point for changing or trying to change and control the lifestyle habits*.” (Interviewee 8, Mother)

“*I think what the speaker said was practical. I saw substantial effects in children. They became willing to get off the bus one station before the destination to walk more. Previously they asked if they could take a taxi when going out on hot days. Now they offered to walk. They could persist. The effects could be seen.”* (Interviewee 6, Mother)

Strong emphasis was placed on parent-child co-activity throughout intervention design and implementation. When parents were asked about the transformation that occurred after participating in the program, the most frequently mentioned point was the improvement in parent-child relationships. Through the program, some parents have begun to reflect on their parenting behaviors and became aware of the importance of establishing PA and co-activity routines.

“*The workshops were highly beneficial to me. As I previously stated, sometimes the parent-child relationship is strained, and I am unsure of what and how to teach my child. Without these classes, I would have continued to discipline my children in my way and demanded more and more of him*.” (Interviewee 15, Mother)

### Appropriate support of environmental factors can enhance the sustainability of program effects

Apart from the characteristics of this PL program fitting the special pandemic circumstance contributed to the sustainability of the program, general environmental factors could also influence the sustainability of PA intervention based on parents' statements.

In terms of the social environment factors, parents expressed positive attitudes and values toward PA. They recognized that regular PA is essential to physical and mental health, and co-activity with children is an important part of their family routine, which helps to strengthen family bonds in regular time. However, parents stated that they did not set up a PA routine for themselves or their children, such as arranging a specific activity at a certain time of day. On weekends, nonetheless, the majority of families frequently participated in outdoor activities together.

Regarding parental efficacy, parents claimed that they have some exercise habits, such as hiking, jogging, cycling, etc. However, they did not identify themselves as “sportspersons.” Moreover, parents expressed that their own PA knowledge and related skills needed to be improved. Despite knowing that parental support was necessary, they lacked confidence in their ability to support their children's PA. Parents also believed that at this stage, children's academic performance is of great importance. Children have to finish their homework first before doing sports or participating in PAs. They also expressed the difficulties in balancing homework and PA time for their children. One mother stated:

“*In Hong Kong, it is challenging to find time to play with children. Although children are not at school the whole day, they have to take online classes when they get home, and after school, it is nearly 4:00 pm, and then they must do their homework. They have to prepare for exams or do other revisions over weekends, making it difficult to find time to participate in PA.”* (Interviewee 6, Mother)

In terms of the physical environment in the local context, almost all parents stated that their living space is relatively small, with insufficient space for PA indoors. Some parents considered the community has a relatively good physical environment and sufficient facilities and space for children to exercise.

“*In the community, the parks around are convenient [for doing PA]. But the home environment may not be spacious enough, for example, for a four-person family like us.”* (Interviewee 11, Mother)

However, some other parents have expressed concerns that although some sports venues are free to the public, in practice, it is frequently impossible to book venues due to high demand. Besides, parents also stated that the distribution of sports resources varies by district.

“*Generally, I think there is not enough support [from the community and school] to support parent-child co-activity. We have tried but failed to book badminton courts. The chance to succeed is zero.”* (Interviewee 7, Mother)

“*If you want to do sports, such as riding a bike, you must travel to a different district that is further away*.” (Interviewee 3, Mother)

Though the current program satisfied participants' needs of doing PA at home during the pandemic, in the long term, for the sake of the sustainability of this and other programs, the social and physical environment factors need to be concerned and improved by researchers and policymakers.

## Discussion

This study explored participants' perceptions of a parent-focused PL intervention and facilitators and barriers to physical activity in the family context before and during the COVID-19 pandemic. Our results suggested that intervention on parents' PL was feasible and sustainable in supporting both parents and children to establish physically active routines in the family setting.

### Acceptability of the program under the COVID-19 pandemic

The purpose and design of this parent-focused PL program were well-matched with the situation of the COVID-19 pandemic. Both parents and children became physically inactive upon the influence of the COVID-19 (21, 22). Particularly, schools and many workplaces were closed, resulting in transitioning activities that once took place outside of the home to virtual implementation in the home environment. For children, this transition meant adapting to online learning without physical encounters with teachers, friends, and a supportive school environment. However, under these circumstances, the marginalization of school physical education has become even more evident (23). As such, PA support provided by parents have become even more important.

For many parents, stay-at-home orders meant they had to navigate their work responsibilities and home chores, assist children with online learning, and juggle the stress of an indefinite pandemic at home. Apart from the potential stress associated with handling daily activities of children, parents' anxiety toward the pandemic may also negatively impact their tendency of allowing their children engage in outdoor activities (24). The provision of a timely education or reminder on the importance of PA is therefore of great importance. As a response, our PL workshops were designed to improve parental knowledge and skills and enhance PL among parents and children. Parent education training sessions may have a favorable impact on parenting practices related to PL. In the current study, parents expressed that their PL was improved and consciously carried out parent-child activities. As such, this finding of the study holds promise, given the potential scalability of the program.

The high levels of acceptability were attributable to the fact that parents were able to learn and be a gatekeeper to impact their children positively. Moreover, the design and contents of the program were appropriate for the participating parents because the program provided adequate learning resources, interactive learning support without being overly rigid, and the online learning mode is feasible, efficient, convenient, and offers parents the accessibility of time and place. The current program is not a one-time intervention but a gradual intervention in 3-month. Unlike other studies (25), the PL knowledge was delivered in several workshops rather than one single session. This may help parents to take in the knowledge step-by-step and be reminded of the intervention regularly. In the long run, parents expressed that the program was meaningful as they became more aware of the importance of PL, gained essential knowledge and skills, established PA routines, and allowed them to reflect on their parenting styles and further improved parent-child relationships. All these should facilitate the sustainability of the intervention afterward.

In addition, the online learning mode is feasible. Under the effect of the COVID-19 pandemic, delivering the intervention *via* the online real-time mode can avoid direct interaction between people during the pandemic. Furthermore, online learning is more adaptable, flexible, and comfortable for parents compared to face-to-face engagement (25). Most parents benefit from online learning because it provides an alternate exercise opportunity. It can be challenging to require parents to commit to attending the face-to-face sessions regularly when they have hectic day-to-day work and lives. However, delivering the program in a feasible online manner can increase their motivation to participate. To some extent, the online training mode is not only applicable to the current pandemic situation but also promising for future implementation of parent education programs.

### Parents' recognition and improvement of PL

According to the SCT, this study adds to the existing literature by probing into parental perceptions and activities that may facilitate or impede children's PA in behavioral, cognitive, and environmental contexts (15). Many parents had favorable attitudes toward the PL program. They recognized the benefits of parental PA support and considered it necessary, consistent with prior research (26). Parents underlined the benefits of PA involvement in terms of physical and mental health and the psychosocial implications. Parents especially enjoyed the interactive activity sessions led by members of the research team. Fundamental movement skills, physical fitness, home-based PAs, and parent-child activities were all covered in this engaging activity program. These parent education sessions provided parents with creative ideas of using their leisure time or living space. Making full use of available environmental resources, such as doing PA with their children together at home. This has been shown to subsequently influence children's PA behaviors favorably and may also help parents and children connect more actively. As PL is a multifaceted construct of physical, cognitive and affective area, the improvement of parents' physical literacy not only facilitate their PA (27) and physical health but also facilitate their children's (27, 28). In the current program, some parents and their children form habits of doing PA in daily life by following instructions in workshops. We hope that *via* parents' modeling and instructions, children's PL will be improved (6, 7). This would also help improve or sustain the effect of family-based or even school-based physical activity interventions in the family context (12).

### Environmental factors influencing PA engagements

Participants' perceptions may provide insights into sustaining PA interventions after the pandemic for researchers and policymakers. Parents identified general barriers, i.e., social and physical environments, to PA engagement, such as exhaustion, lack of time and family support, inclement weather, and the Hong Kong built environment, which are consistent with earlier studies (29–31). Most importantly, in keeping with previous findings (32), the participating parents often lack prior education and training in PL and other aspects of their sports parenting skills. Although they recognize the benefits of PA, they have insufficient basic knowledge and skills to support them in establishing a PA routine. Given that sports parenting is more complicated and influenced by various factors when attempting to encourage parents' participation in PA, it cannot be the case that parents are simply informed what they should or should not do; instead, it is crucial to understand and remove their obstacles to facilitate a PA-friendly environment. For example, researchers should deliver public lectures on PL to families. Policymakers should improve PA facilities in communities.

### Strengths and limitations

To our knowledge, this is the first study to explore the acceptability of a parent-focused PL intervention. In this regard, we sought to determine the program's acceptability in the context of the COVID-19 pandemic and grasp the subsequent influence on parents' and children's lives and draw lessons for the future. Besides, a major strength of the current study is that it helps to address the critical need for more work focused on parents' experiences to inform the implementation of parent education training sessions in diverse settings. Moreover, parent workshops and materials were delivered *via* online mode. This is an exploratory attempt to understand the uniqueness and acceptability of online learning and training in the impact of the current pandemic and future practice. Another strength of the study is its rigorous methodology, including a theoretically-informed interview guide and preliminary codebook, inductive-deductive hybrid thematic analysis, two coders' independent coding, and member checking process. Lastly, family-based interventions showed mixed effects. Some showed small to moderate effects whereas some showed no effects (33, 34). We hope the results of this study could help to understand why some interventions are not successful in the family context. For example, parents might not be aware of the purpose or importance of PA interventions so no parental support may not be provided after the intervention (34).

Some limitations should be considered. One limitation is that almost all participating parents were mothers. Although we invited all the parents who had participated in the parent-led program to attend, we still lacked fathers' participation. Since mothers and fathers may have different experiences and perspectives of the program, future research could explore more about fathers' perceptions. Furthermore, to have a more holistic understanding of the implementation process and the impact on each family, further work is needed to explore the program's sustainability and quantitatively evaluate the behavioral change of participants to inform the expansion of parent-led programs.

## Conclusions

This study explored parents' perceptions of a parent-focused PL program during the COVID-19 pandemic. Parents' PL was generally improved from the learning experience and the commitments that came with establishing physically active lifestyles in the family setting. Some of their children's PL was also improved. The parent-focused PL program strongly emphasizes parent role modeling and co-participation in PA, making it the ideal candidate for long-term implementation *via* online delivery. The insights gained about the program of acceptability in this specific context, and general environmental factors influencing participants' PA, may be used to inform the design, implementation and sustainability of other parent-focused PL programs.

## Data availability statement

The raw data supporting the conclusions of this article will be made available by the authors, without undue reservation.

## Ethics statement

The studies involving human participants were reviewed and approved by Survey and Behavioral Research Ethics Committee of the Chinese University of Hong Kong. Written informed consent to participate in this study was provided by the participants' legal guardian/next of kin.

## Author contributions

AH, QH, CC, and JN conceived of, designed, and supervised the study. TZ and QH conducted the analysis. TZ drafted the manuscript with input from QH, YF, JN, and AH. TZ and JN revised the manuscript. All authors contributed to the interpretation of data, revision of the manuscript for important intellectual content, and have read and approved of the final version of the manuscript.

## Funding

This research was supported by the Hong Kong Jockey Club Charities Trust.

## Conflict of interest

The authors declare that the research was conducted in the absence of any commercial or financial relationships that could be construed as a potential conflict of interest.

## Publisher's note

All claims expressed in this article are solely those of the authors and do not necessarily represent those of their affiliated organizations, or those of the publisher, the editors and the reviewers. Any product that may be evaluated in this article, or claim that may be made by its manufacturer, is not guaranteed or endorsed by the publisher.
